# Long term outcomes from lymphatic venous anastomosis after total hysterectomy to prevent postoperative lymphedema in lower limb

**DOI:** 10.1186/s12893-019-0628-z

**Published:** 2019-11-26

**Authors:** Masahiro Ezawa, Hiroshi Sasaki, Kyosuke Yamada, Hirokuni Takano, Tsuyoshi Iwasaka, Yoshifumi Nakao, Tomoki Yokochi, Aikou Okamoto

**Affiliations:** 1 Department of Obstetrics and Gynecology, The Jikei University School of Medicine, Tokyo, Japan; 2 Department of Gynecology, Chiba Tokushukai Hospital, Chiba, Japan; 3 Department of Obstetrics and Gynecology, Saga University School of Medicine, Saga, Japan; 4 Preventive Medical Center, Takagi Hospital, Fukuoka, Japan; 5 Department of Clinical Research, Chiba Tokushukai Hospital, Chiba, Japan

**Keywords:** Lymphedema, Microsurgery, Lymphaticovenous anastomosis

## Abstract

**Background:**

Lymphedema in lower limb is one of major postoperative complications followed by a total hysterectomy with lymph node dissection. The objective of this report is to examine a long-term result of lymphaticovenous anastomosis procedure as a preventive surgery.

**Methods:**

Sixteen patients with endometrial cancer underwent an abdominal hysterectomy with a bilateral salpingo-oophorectomy. Just after pelvic lymph node dissection, either end-to-end or sleeve anastomosis utilizing venules and suprainguinal lymph vessels was performed. During the observation period from 4 to 13 years, the symptom of lymphedema in lower extremities has been assessed.

**Results:**

Among 16 patients, 1 presented postoperative lymphedema grade 3 (CTCAE (Common Terminology Criteria for Adverse Events) Ver. 4.0, 10025233) in lower limb, and a second surgery at 7 years after the first one was required. Other 6 patients showed non-severe symptoms of lymphedema, diagnosed as grade 1. The rest 9 patients did not show any symptoms of postoperative lymphedema in a long term (up to 13 years).

**Conclusion:**

From the long term outcomes of our 16 cases, we propose that a direct lymphaticovenous microsurgery immediately after a hysterectomy with lymphadenectomy of external inguinal lymph node is one of the appropriate therapeutic choices to prevent severe lymphedema in lower limb.

## Background

Lymphedema is a disease condition in which extracellular fluid is aberrantly accumulated in the soft tissue beneath the skin. As one of major postoperative complications in the patients with gynecological malignancies in which lymph nodes are dissected, lymphedema in lower limb significantly decreases the quality of life of patients for a long period. Incidence rates of lymphedema in lower limb have been reported to be 1.2–68% variation in Japan, which greatly depend on potential risk factors, such as radiation and the site of lymph nodes dissected [1–6].

As one of the measures to improve lymphedema, lymphaticovenous anastomosis procedure has been reported [7]. Conventionally, the lymphatic venous anastomosis has been performed for the postoperative treatment of lymphedema in either upper or lower limbs after surgery, while the cure rate varies [8–11]. For one reasons of this variation, it was reported that edema-improving effect is deteriorated even if the anastomosis is performed, because structure of lymph duct has been destroyed and thus reflux of the lymph fluid to the vein is not improved. Furthermore, the progression of lymphedema reduces the efficacy of lymphatic venous anastomosis because of fat hypertrophy, fibrosis, and induration [12]. In that case, however, it was expected that lymphedema in lower limbs can be effectively improved by the lymphatic venous anastomosis particularly at the time when the lymphatic vessel function has not been destroyed, for example, immediately after the lymphadenectomy [9, 13].

Therefore, the primary objective in this study is to evaluate the clinical characteristics and long term outcomes of the lymphatic venous anastomosis just after the lymphadenectomy in order to prevent lymphedema in lower limbs. This procedure may be useful to improve the effect of lymphedema, as the anastomosis between lymph ducts and venous may be successful to reduce the leak of the lymph fluid. However, there are few literatures previously describing the improving effect for lymphedema. The aim of this report is to examine a long-term result of the lymphaticovenous anastomosis procedure as a preventive surgery against lymphedema.

## Methods

### Baseline characteristics

Subjects of this study included a total of 16 patients with endometrial cancer, which were histologically confirmed. All cases underwent dissection of more than 20 lymph nodes in the period between September 2003 and August 2011. Exclusion criteria were the patients with double cancer, distant metastasis, or existing lymphedema. Medical records of the 16 patients were retrospectively reviewed. Observation period of the patients was between 4 and 13 years, as of 2018.

### Surgical procedure

In the surgical treatment of endometrial cancer patients, a total abdominal hysterectomy with a bilateral salpingo-oophorectomy was performed. After a pelvic dissection of lymph node in paraaortic and extra inguinocrural regions, the retroperitoneum was closed except in the pelvis. Immediately after the surgery, an operating microscope was brought in to identify venules with a diameter of 1.5 mm arising from the inferior epigastric vein on the abdominal wall at inguinal region. Approximately 1 cm length of the venula was picked up. Also, the largest part of a lymph vessel connecting suprainguinal lymph nodes was carefully identified as a recipient utilizing methylene blue dye, where lymph flow is consistent [14, 15]. Lymphaticovenous anastomosis was performed utilizing the venula and lymph vessel, while other free lymph vessels were tied and closed. When the diameters of the venula and lymph vessel are the same in length, end-to-end anastomosis was conducted. When the lymph vessel picked up was too narrow for end-to-end anastomosis, sleeve anastomosis was performed [16]. The same procedure was repeated for the internal iliac lymphatics located near the internal iliac vein. This procedure was performed for each of the lymphatic-venous pairs located adjacent to the branch of lower abdominal veins (two anastomosis or four anastomosis) on the left and/or right sides. In all cases, the physician or operating nurse explained to the patients and agreed on the time and course of the procedure [17].

### Postoperative procedure

This study focused on symptomatic lymphedema in the lower limb. Essentially, swelling of lower extremities was diagnosed on the basis of information provided by patient and the international classification regarding the severity of lymphedema [18]. The diagnosis was confirmed by multiple methods including MRI (Magnetic Resonance Imaging), CT (Computed Tomography), and ultrasonic imaging. The diagnostic criteria for lymphedema was done by comparison of the legs on both side and pitting edema. The onset of lymphedema was determined by measuring the girth of patient’s limb. The 11 out of 16 patients received postoperative chemotherapy, while none of them had radiotherapy. All cases with postoperative lymphedema received compression stockings.

## Results

Suprainguinal lymphaticovenous anastomosis was performed on 16 cases of the patient. Patient characteristics are summarized in Table 1. All patients underwent pelvic- and para aortic-lymph node dissection, accompanied by a total abdominal hysterectomy with a bilateral salpingo-oophorectomy. The range of patient’s age was from 35 to 73 years old and the mean was 48. Performance status of all patients was zero. The numbers of the patients in each clinical stage of endometrial cancer were: stage I, 10 cases; stage II, 1 case; and stage III, 5 cases. Tumor histology indicated carcinosarcoma in 1 case and endometrioid adenocarcinoma in 15 cases. Adjuvant chemotherapy was used in 11 cases and hormone therapy after surgery was done in 2 cases (data not shown).

**Table 1:** Patient characteristics

Case (Facility)	Staging of cancer	BMI	Histology	Postoperative chemotherapy	Survival and observation period (Month)
1 (I)	III A	23.6	CS	IAP	ANED at 102
2 (I)	I A	23.2	EAD Grade1	MPA	ANED at 65
3 (I)	III C1	19.6	EAD Grade2	AP	ANED at 96
4 (I)	I A	22.2	EAD Grade1	MPA	ANED at 157
5 (I)	I A	20.3	EAD Grade1	-	ANED at 127
6 (I)	III C1	20.8	EAD Grade3	AP	ANED at 94
7 (I)	I A	23.4	EAD Grade3	AP	ANED at 163
8 (I)	I A	20.3	EAD Grade1	-	ANED at 154
9 (II)	III C1	20.3	EAD Grade1	TC	Deceased at 115
10 (II)	I A	18.4	EAD Grade3	-	ANED at 136
11 (II)	II	19.6	EAD Grade1	DC	ANED at 65
12 (II)	I A	22.6	EAD Grade2	-	ANED at 120
13 (II)	I B	19	EAD Grade1	DC	Deceased at 118
14 (II)	I A	26.5	EAD Grade1	-	ANED at 62
15 (II)	III A	19.6	EAD Grade2	DC	ANED at 49
16 (II)	I B	19.7	EAD Grade2	DC	ANED at 77

Facility (I): The Jikei University, (II): Saga University; CS: Carcinosarcoma; EAD: Endometrioid adenocarcinoma; IAP: Ifosfamide, farmorubicin, cisplatin; MPA: Medroxyprogesterone acetate; AP: Adriamycin, cisplatin; TC: Paclitaxel, carboplatin; DC: Docetaxel, carboplatin; ANED: Alive with No Evidence of Disease regarding cancer

Detailed surgical summary of lymphedema is shown in Table 2. The surgical procedure described above was completed on both right and left sides in 14 cases out of 16. It should be noted that a total of four venous-lymphatic vessel anastomoses were performed by two at each side in the Jikei University (Cases 1–8, facility I), while a total of two anastomoses were done by one on each side in Saga University (Cases 9–16, facility II). Operative time ranged from 7 to 11 h. During the anastomosis, procedure-specific bleeding was not observed. Indeed, reddish lower limbs, which may indicate traces of blood in the lymph vessels, were not yielded immediately after surgery in any case. There was no significant difference between these two facilities, and the long-term results were similar in regards to the staging of lymphedema grade I (Facility I / II = 2/8:4/8).

**Table 2:** Clinical characterizations of lymphedema

Case (Facility)	Type of Anastomosis		Grade of lymphedema on CTCAE (v.4.0, 10025233)		Timing of the appearance of lymphedema (Month)	Operation time		Cormobidity
	Right suprainguinal	Left suprainguinal						
	External / Internal	External / Internal	Right leg	Left leg		Total time (hour:min)	Gynecology / Plastic surgery (min)	
1 (I)	E to S / E to E	E to E / E to E	1	0	2	10:00	330 / 270	-
2 (I)	E to E / E to E	E to E / E to E	0	0	-	08:45	285 / 240	-
3 (I)	E to E / E to E	E to E / Sleeve	0	0	-	11:00	400 / 260	Aneurism at 38 years old, cured
4 (I)	E to E / E to E	E to E / E to E	0	0	-	10:10	370 / 240	-
5 (I)	E to E / E to E	E to E / E to E	0	0	-	10:30	375 / 255	Tuberculosis at 25 years old, cured
6 (I)	E to E / E to E	E to E / E to E	3	3	38	10:00	360 / 240	Asthma
7 (I)	E to E / E to E	E to E / Sleeve	0	0	-	09:15	375 / 180	-
8 (I)	E to E / Sleeve	E to E / E to E	0	1	0	08:40	385 / 135	Fibroid removal at 35 years old
9 (II)	E to S / -	- / -	0	0	-	09:40	375 / 205	-
10 (II)	E to S / -	E to S / -	0	0	-	09:00	380 / 160	Gilbert’s syndrome
11 (II)	E to S / -	E to S / -	0	1	26	08:00	375 / 105	-
12 (II)	E to S / -	E to S / -	0	1	15	08:50	415 / 115	-
13 (II)	- / -	E to S / -	0	0	-	07:30	290 / 160	Hypertension, breast cancer
14 (II)	E to S / -	E to S / -	0	0	-	10:00	410 / 190	-
15 (II)	E to S / -	E to S / -	0	1	4	07:40	375 / 85	-
16 (II)	E to S / -	E to S / -	1	0	24	08:00	320 / 160	-

E to S: End to Side lymphaticovenous anastomosis, E to E: End to End lymphaticovenous anastomosis, Sleeve: Sleeve anastomosis. CTCAE: Common Terminology Criteria for Adverse Events (Version 4.0, MedDRA V12.0 code: 10025233)

**Fig. 1:**
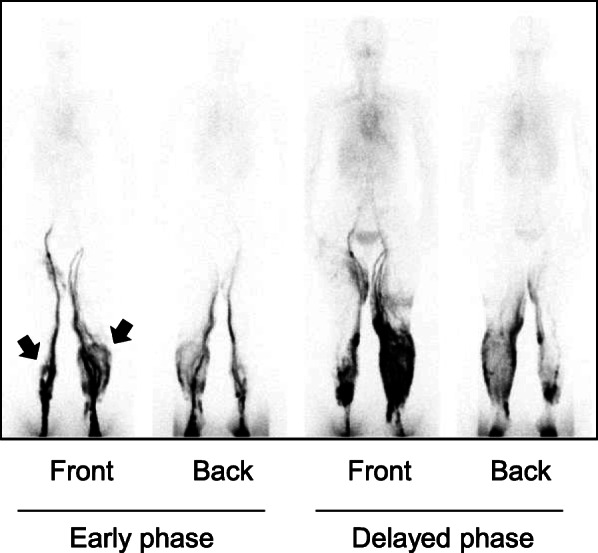
Lymphangiography of Case #6 at 7 years and 3 months (87 months) of post-surgery. From front and back side images were shown in lymphatic scintigraphy (740 MBq) in early (left) and delayed (right) phase. MRI contrast agent (tracer) stayed in bilateral lower leg, indicating lymphedema. Note that visualization above the pelvic region is less clear. The arrow indicates grade III lymphedema in the patient

Long-term outcomes of the lymphaticovenous anastomosis procedure to prevent postoperative lymphedema were examined for up to 13 years. Postsurgical lymphedema was observed in 6 cases, in which symptoms were relatively mild with a diagnosis of grade 1 (CTCAE v4.0, Table 2). One patient presented lymphedema at 7 years and 3 months (87 months) after surgery, diagnosed as grade 3 (Fig. 1). She had repeated remission and exacerbation of lymphedema a couple of times, after resolving bilateral cellulitis caused by a tick bite at postsurgical 6 years and 2 months (74 months). At 7 years and 0 months (84 months), we diagnosed her with exacerbation of lymphedema in the lower limb. At 7 years and 3 months, lymph duct scintigraphy was performed (Fig. 1), and then lymphatic venous anastomosis was conducted at right lower limb at 7 years and 5 months (89 months). All patients are alive and no evidence of recurrence has been observed during the last 5–10 years.

## Discussion

The risk of lymphedema is higher in the presence of several risk factors such as obesity, physical inactivity, and venous insufficiency. Thus, preventive approaches are generally applied to this increased risk subset of patients [19–21]. In this study, we have demonstrated that lymphaticovenous anastomosis provides one of the effective procedures to prevent lymphedema in lower limbs after a total hysterectomy with lymph node dissection. It should be noted that anastomosis between the lymph vessel and venula has to be performed immediately after pelvic lymph node dissection, yielding preservation of the structure of the lymph vessel.

In the previous report, there is a large gap in the frequency of lymphoedema of 1.2–39.1% [6, 22]. One of the reasons for this is that the frequency of lymphoedema decreases by leaving the external inguinal lymph nodes [2, 6]. Also, the reason why the subject of this study is only uterine body cancer is that the external inguinal lymph node is the primary regional one and thus the dissection cannot be omitted. The position connecting the lymph vessel and the blood vessel is where the external inguinal lymph node is located. So far, there was no effective way to reduce the frequency of lymphoedema in cases where the external inguinal lymph node had to be taken. According to the result of Todo et al., lymphedema at lower limb was less than 26.5% when the external inguinal lymph node was left [2, 6, 22]. In our procedure, adverse events more than grade 2 was 6.5% (1/16), and thus it was almost comparable to Abu-Rustum’s report [23]. Thus, our anastomosis procedure is useful when we have to take lymph nodes at lower limb. We performed anastomosis at the site of the external inguinal lymph nodes for two reasons. First, damage or ruptured suture might not occur when the patient moves his/her legs after surgery, because the load is not applied to the anastomotic part. Second, dissection at the external inguinal lymph nodes may cause severe lymphedema [2, 6].

Several risk factors for postoperative lymphedema in lower limb have been reported [1, 2]. For instance, adjuvant radiotherapy increased the incidence of lymphoedema of the legs following pelvic lymphadenectomy [24, 25]. In our study, however, none of patients had radiotherapy and thus it remains less conclusive. Alternatively, a wide range of lymphadenectomy may be responsible for increasing the risk of the lower limb lymphedema. It is necessary to discuss whether lymph node dissection in a total hysterectomy has to extend to even paraaortic region in addition to pelvic region.

It is assumed that direct lymphaticovenous anastomosis followed by a hysterectomy and lymphadenectomy is effective to prevent postoperative lymphedema. This approach would not only reduce the risk of lymphedema, but also reduce the risk of lymphocele [26, 27]. Potential problem we recognized at surgery, however, is that surgical results significantly depended on each surgeon using a microscope. Since microsurgery is a typical technique-oriented approach, it may take a long time to establish a skilled physician. Indeed, we have seen that each plastic surgeon had the difference in microsurgical technique. From this standpoint, lymphaticovenous anastomosis employing da Vinci Surgical System (Intuitive Surgical, Inc., CA) may be a reasonable and feasible mean of solving. Stable movements of robot arms in the da Vinci system allow the surgeon to operate with enhanced vision, precision, and control in lymphaticovenous anastomosis. In our facility, this approach as an advanced medical treatment has been approved by the Ministry of Health, Labor, and Welfare of Japan and is ongoing. The result of this clinical trial will be published elsewhere.

## Conclusion

From the long term outcomes of our 16 cases, we concluded that a direct lymphaticovenous microsurgery immediately after a hysterectomy with lymphadenectomy of external inguinal lymph node is one of the appropriate therapeutic choices to prevent severe lymphedema in the lower limb.

## Data Availability

The datasets used and analyzed during the current study were available from the corresponding author on reasonable request.

## Abbreviations

ANED: Alive with No Evidence of Disease regarding cancer
AP: Adriamycin, cisplatin
CS: Carcinosarcoma
CT: Computed Tomography
CTCAE: Common Terminology Criteria for Adverse Events
DC: Docetaxel, carboplatin
E to E: End to End lymphaticovenous anastomosis
E to S: End to Side lymphaticovenous anastomosis
EAD: Endometrioid adenocarcinoma
IAP: Ifosfamide, farmorubicin, cisplatin
MPA: Medroxyprogesterone acetate
MRI: Magnetic Resonance Imaging
Sleeve: Sleeve anastomosis
TC: Paclitaxel, carboplatin
